# Saccadic Performance and Cortical Excitability as Trait-Markers and State-Markers in Rapid Cycling Bipolar Disorder: A Two-Case Follow-Up Study

**DOI:** 10.3389/fpsyt.2012.00112

**Published:** 2013-01-04

**Authors:** Jennifer Malsert, Nathalie Guyader, Alan Chauvin, Mircea Polosan, David Szekely, Thierry Bougerol, Christian Marendaz

**Affiliations:** ^1^CNRS, UMR 5105, Psychology and NeuroCognition LaboratoryGrenoble, France; ^2^CNRS, UMR 5216, Grenoble Image Parole Signal Automatique LabGrenoble, France; ^3^Department of Psychiatry, University HospitalGrenoble, France; ^4^INSERM, U836, Grenoble Institut des NeurosciencesGrenoble, France

**Keywords:** TMS, saccade, cortical excitability, paired-pulse, inhibition/facilitation, GABA/glutamate

## Abstract

**Background:** The understanding of physiopathology and cognitive impairments in mood disorders requires finding objective markers. Mood disorders have often been linked to hypometabolism in the prefrontal dorsolateral cortex, and to GABAergic and glutamatergic neurotransmission dysfunction. The present study aimed to discover whether saccadic tasks (involving DPLFC activity), and cortical excitability (involving GABA/Glutamate neurotransmission) could provide neuropsychophysical markers for mood disorders, and/or of its phases, in patients with rapid cycling bipolar disorders (rcBD). **Methods:** Two rcBD patients were followed for a cycle, and were compared to nine healthy controls. A saccade task, mixing prosaccades, antisaccades, and nosaccades, and an evaluation of cortical excitability using transcranial magnetic stimulation were performed. **Results:** We observed a deficit in antisaccade in patients independently of thymic phase, and in nosaccade in the manic phase only. Cortical excitability data revealed global intracortical deficits in all phases, switching according to cerebral hemisphere and thymic phase. **Conclusion:** Specific patterns of performance in saccade tasks and cortical excitability could characterize mood disorders (trait-markers) and its phases (state-markers). Moreover, a functional relationship between oculometric performance and cortical excitability is discussed.

## Introduction

Affective disorders are the most disabling of neuropsychiatric conditions, and one of the four leading causes of disability [World Health Organization (WHO), 2003]. Acquiring a better understanding of the physiopathology and cognitive impairments in mood disorders requires the finding of “objective” indicators of the illness. In the present pilot study, we tested two tools: oculometry (saccadic tasks) and cortical excitability (via magnetic transcranial stimulation of the motor cortex), using a particular clinical paradigm: the follow-up of two patients suffering from rapid cycling bipolar disorders (rcBD). The experimental rationale was as follows.

### Mood disorders and saccadic performance

Over the past three decades, there has been an increase in the number of neuropsychophysical studies of saccadic performance in psychiatric patient groups (Gooding and Basso, 2008). Much of the impetus for the focus on saccadic eye movements in these populations comes from the fact that saccades provide a non-invasive yet accessible means of investigating psychomotor functioning as well as higher-order cognitive processes and their underlying neural mechanisms. Most of the studies have focused on schizophrenia patients, using mainly the antisaccade (AS) task (Hallett, 1978). In AS, subjects have to inhibit a reflexive movement toward a peripheral cue and to produce a voluntary saccade in the opposite direction (mirror position). In this task, the variables of interest are: saccade errors, also known as inhibition errors (saccades toward the cue); and latencies (or saccadic reaction time, SRT) for correct AS. Fukushima et al. (1988) showed increased proportions of saccade errors during AS in schizophrenic patients, a result replicated many times and associated with reduced metabolism in the frontal lobes (McDowell et al., 2002) or reduced frontal brain volumes (Fukushima et al., 1990a). In schizophrenia, AS performance is one of several endophenotype candidates (Radant et al., 2007). While the number of studies on eye movements and patients with schizophrenia or schizophrenia-spectrum disorders is huge, there is relatively little research on eye movements and mood disorders. In early investigations of saccadic performance, the latter type of patients were regarded primarily as psychiatric control subjects and were mixed into a single group regardless of their pathologies (bipolar or unipolar). Investigations of AS performance in patients suffering from major depressive disorder (MDD) indicate that mild or moderately depressed patients display normal rates of inhibition error saccades (Fukushima et al., 1990b), while acutely ill, unmedicated depressive patients display elevated saccade error rates (Harris et al., 2009) or increased AS latencies (Crevits et al., 2005). These findings suggest, on the whole, that AS impairments in major depression may be state-related. A better way to characterize the relationship between mood state and saccadic performance is to examine bipolar patients switching between opposing depressive and manic phases. To date, there have been few published reports concerning bipolar disorders and saccadic performance, and only one (Gooding et al., 2004) has followed bipolar patients. In Gooding et al. (2004), 33 schizophrenia out-patients and 10 bipolar out-patients performed two saccadic tasks (pro and AS) at two separate assessments, with an average delay of 33 months. Results showed that schizophrenic patients displayed high test-retest reliabilities in the AS task despite changes in medication and clinical status. By contrast, bipolar patients did not show temporal stability in their AS performance. It is, however, worth noting that the symptomatic status of bipolar patients also changed over time. At the time of their initial assessment, the clinical status of bipolar patients was mixed; three were depressed, two were euthymic, and five were hypomanic. At the time of retest, nearly all patients (90%) were acutely symptomatic; six were depressed, and three were hypomanic. These clinical switches from test to retest might be the reason why AS performance was unstable between the two assessments. Unfortunately the authors did not analyze intra-individual saccadic performance between illness phases. Does saccadic performance change according to mood phase? Therefore as far as we know, no study has compared saccadic performance between depressive and manic phases. In this study, we followed two patients with bipolar disorder during the different phases of their illness using a mixed SPAN (Saccade Pro, Anti, and No) task. Parallel measures of cortical excitability were also carried out.

### Mood disorders and cortical excitability

The use of transcranial magnetic stimulation (TMS) as a non-invasive method of inducing depolarization or hyperpolarization in neurons allows researchers to study functioning and interconnections of the brain. Applied to the motor cortex, the TMS magnetic field provides an opportunity to evaluate not only cerebral functional activity but also neurochemical properties, because the magnetic coil preferentially depolarizes horizontally oriented axons in the cortex. These axons consist primarily of excitatory (glutamatergic) and inhibitory (GABAergic) interneurons which regulate local excitability via very short axons (Markram et al., 2004; Kapogiannis and Wassermann, 2008). This technique gives a global view, known as “cortical excitability,” of interneuron activation in the cerebral cortex, and consequently any possible deficit in one of them. Cortical excitability measures consist of the study of neuronal reactivity in terms of resting motor threshold (RMT), baseline, intracortical inhibition (ICI)/facilitation (and sometimes the cortical silent period) from the TMS pulse through amplitude recordings of motor evoked potentials (MEP; see Materials and Methods).

Mood disorders are reflected by impairment principally in the prefrontal cortex, but their clinical presentation suggests that motor deficits are associated with the pathology (Campbell and MacQueen, 2006). Moreover, clinical scales include items on motor retardation; it is thus coherent to evaluate motor excitability as an indicator of the neurophysiopathology underlying mood disorders. Samii et al. (1996) observed a decrease in post-exercise facilitation in depressive vs. control subjects, corresponding to a deficiency in cortical excitability. These results were confirmed by Shajahan et al. (1999), who suggested that facilitation correlated with depression severity and recovered after remission, corresponding to a state indicator. Cortical excitability measured in each hemisphere can also be used to study interhemispheric asymmetries. Indeed, several neuroimaging studies have suggested that MDD correspond to a disturbance in cortical activity with lower left dorsolateral prefrontal cortex (DLPFC) or higher right DLPFC activity (Baxter et al., 1989; Davidson and Meltzer-Brody, 1999). TMS studies showed a higher RMT in the left hemisphere than in the right, in patients with MDD (Maeda et al., 2000). This observation suggests stronger right cortex excitability in patients, not noted in control subjects and consistent with neuroimaging. Another study presented a decrease in right RMTs in patients, corresponding to right-sided hypermetabolism (Bajbouj et al., 2006a). In bipolar disorders, Levinson et al. (2007) demonstrated a significant deficit in cortical inhibition in patients compared to controls. As cortical inhibition processes are under the control of GABAergic interneurons, these results show GABAergic pathway involvement in depressive pathologies. The one longitudinal study in bipolar patients was led by Chroni et al. (2008). The authors evaluated the post-exercise facilitation of MEP in two patients during different phases of rapid cycling depressive-manic disorder. Post-exercise MEP facilitation was significantly lower than control values, but no differences were revealed between depressive and manic phases, suggesting an invariable underlying association between physiopathological psychiatric mechanisms and impaired cortical excitability. To sum up, cortical excitability seems to be a plausible biological correlate of disease and recovery in mood disorders. So, we used this neurophysiological tool to characterize rcBD and its phases, and to gain better understanding of some of the possible deficits in saccadic performance.

### Pilot study aims

The research aimed to test whether saccadic performance and cortical excitability measures could “sign” (be indicative of) rapid cycling BD disease *per se* (trait-marker) and/or its phases (state-marker), and if so, what the functional relationship between the two types of markers might be.

## Materials and Methods

### Participants

#### Patients

Two patients with rapid cycling bipolar disorders were followed during the three illness phases. The diagnosis (Table 1) of rapid cycling bipolar disorder was confirmed in accordance with DSM-IV criteria by using the axis I disorders structured interview (SCID-I, First, 1995), and the clinical severity of the phases of the bipolar disorder were assessed using the Young Mania Rating Scale (Young et al., 1978), and the Montgomery and Asberg Rating Scale (Montgomery and Asberg, 1979).

**Table 1:** **Patient information**.

P1				P2			
Thymic phase	Psychometric scores		Medication	Thymic phase	Psychometric scores		Medication
	MADRS	YMRS			MADRS	YMRS	
Manic	8	20	LTG; CMZ	Depressive	21	0	Li; VPA; VFX
Euthymic	12	4	LTG	Euthymic	8	6	Li; VPA; VFX
Depressive	28	0	LTG; CMZ; S-Cit	Manic	4	22	Li; VPA; OLZ

*Abbreviations: MADRS, Montgomery and Asberg Depression Rating Scale; YMRS, Young Mania Rating Scale; LTG, lamotrigine; CMZ, cyamemazine; S-Cit, escitalopram; Li, Lithium; VPA, divalproex; VFX, venlafaxine; OLZ, olanzapine*.

The first patient, P1, is a 53-year-old right-handed man with a significant severe thymic family history (including two suicides). He experienced his first major mood episode (depressive polarity) at the age of 38, and a very rapid form of mood fluctuations progressively appeared, with 3 week hypomanic episodes and 1–4 week depressive episodes, with subsequent shortening of the inter-critical periods and rapid onset of the acute phases.

The second patient, P2, is 60-year-old and right-handed, with a family history of mood disorders, and has suffered from bipolar disorder since he was 43, with a depressive polarity onset and a rapid evolution toward the rapid cycling form, despite various therapeutic strategies. Patients were evaluated during all three illness phases (2.5 and 1.5 month intervals respectively): Euthymic, Manic and Depressive phases. Test order and measures were counterbalanced between patients.

#### Controls

Nine healthy control participants (four males, mean age 34 ± 11 years) without psychiatric history or medication completed the saccadic task and cortical excitability measures.

All participants met safety criteria for TMS as published by Wassermann (1998) and gave their informed consent to the study approved by the local ethical committee.

### Saccadic performance

#### Apparatus

A camera-based eye-tracker (EyeLink^®^ 1000 from SR Research with EyeLink^®^ CL software) with a temporal resolution of 500 Hz and an accuracy of 0.02° was used, in the pupil-corneal reflection (P-CR) tracking mode. A 3 × 3 point calibration was completed and a drift-correction was carried out every 10 trials. The eyetracker detected saccades automatically as eye movements of a velocity greater than 30°/s and an acceleration greater than 8000°/s^2^. For further analyses we used saccade and fixation events computed by the EyeLink software.

#### Stimuli

Stimuli were displayed on a 21″ mean gray level (10 cd/m^2^ CIE luminance) screen located 57 cm from participants. Screen resolution was 1024 × 768 pixels, and the screen refresh rate 85 Hz. Stimuli were numbers (“0” for the cue and “6” or “9” for the target) written in white (48 cd/m^2^ CIE luminance) inside a 1.2° white ring, appearing peripherally at 10°.

#### Procedure

**Figure 1:**
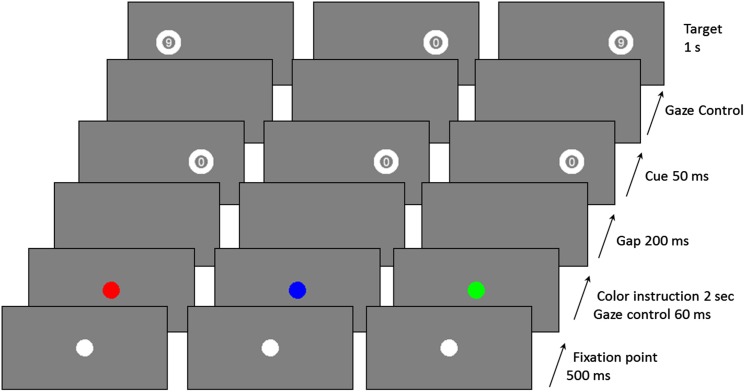
**Examples of trial sequences for the three saccadic conditions (red: AS, Blue: NS, and green: PS)**.

The experimental paradigm is schematized in Figure 1. As mentioned above, it included three saccade types: Prosaccade (PS), AS, and Nosaccade (NS). Patients were tested individually in a darkened room. Head position was stabilized with a chin rest.

The saccadic task depends on the color of the central fixation dot at the beginning of a trial, and uses a gaze contingent display. Participants were told to make a PS if the color was green, an AS if it was red, and a NS if it was blue. In PS condition, participants had to look as quickly as possible at the peripheral cue location to identify the number inside the target and to give an oral response (“6” or “9”). In the AS condition, they had to look as quickly as possible at the opposite side of the cue to identify the number inside the target (which appeared only if participants gazed at the correct location) and give an oral response (“6” or “9”).

Each trial began with a 500 ms presentation of a white central fixation dot. The central fixation dot then became either green, or red, or blue for 2 s. After this time, and only if the participant stared at the fixation dot (±0.5° on the horizontal axis and ±1° on the vertical axis) for 60 ms, a blank screen was displayed for 200 ms. Then, a cue, always the number “0,” was flashed for 50 ms, at 10° peripherally (randomly on the left or right side of the screen). The target appeared as soon as the participant gazed at the correct position (depending on the saccade type; correct location ±1° on the horizontal axis and ±2° on the vertical axis). Otherwise the target appeared after a 2 s delay. The target was presented for 1 s, on the same side as the cue in the PS condition, or on the opposite side in the AS condition, or randomly on the left or right in the NS condition. In the NS condition, participants had to keep their gaze fixed on the centre of the screen. There was a break of 1 s between two successive trials.

The identification task (to identify the number on the target) was chosen to facilitate the understanding of the AS task and make it more pleasant (Guyader et al., 2010). The order of assessments in relation to mood phases was counterbalanced between patients.

Participants underwent a training session of 20 practice trials. In the testing session, there were 80 trials: 16 for the NS condition, 32 for the PS condition, and 32 for the AS condition. The position of the cue (left or right) and the number to be identified (6 or 9) was randomly-distributed across conditions.

### Cortical excitability

We used a TMS MagPro X100 (Medtronic^®^) with a Magoption to deliver paired-pulses. The coil is an eight-coil MCF-B65. We recorded MEP to motor cortex stimulation with three electrodes placed on the contralateral first dorsal inter-osseous, and connected to an electrophysiological recording system (NemusElectramed^®^; FDI). The cortical motor site which produced the largest MEP in the muscle for each hand was located.

The cortical excitability protocol consisted of a set of measures for each hemisphere. Firstly, we estimated the RMT. Then, we measured baseline motor potentials and four paired-pulse protocols (at intervals of 2, 4, 10, and 15 ms). All subjects were tested with the following protocol.

The RMT was determined as the minimum intensity that evoked a peak-to-peak MEP superior to 50 μV in at least five out of 10 consecutive trials. RMT was expressed as a percentage of maximal stimulator Intensity.

To measure the cortical excitability baseline, stimulations at 120% of the RMT were used, and peak-to-peak MEP amplitudes (in μV) were recorded. Ten trials were performed and the five largest were averaged.

The paired-pulse procedure for measuring intracortical processes followed the one described in the literature (Kujirai et al., 1993; Ziemann et al., 1996). All measurements were conducted at rest with continuous MEP recording. An initial conditioning stimulus which did not produce a MEP response was set (at 80% RMT). A second stimulus (test stimulus) used a consistent intensity (120% RMT) that would produce a MEP response. Intracortical effect of conditioning stimulus (inhibitory or facilitatory) is known to vary depending on inter stimulus interval (ISI). This effect was calculated as a percentage of the single pulse baseline, based on the average peak-to-peak MEP size. Ten trials of data were recorded and the five largest responses were averaged. Pharmacological studies (Kujirai et al., 1993; Ziemann et al., 1998) had shown that for ISIs inferior to 6 ms, the conditioning stimulus inhibits the response to the test stimulus, involving GABA_A_ receptors, whereas for ISIs superior to 6 ms, it facilitates responses with NMDA receptors involvement (glutamatergic pathway). To test these two processes, inhibitory and facilitatory, we used four ISI randomly-distributed conditions: 2, 4, 10, and 15 ms intervals.

## Results

### Saccadic performance

**Figure 2:**
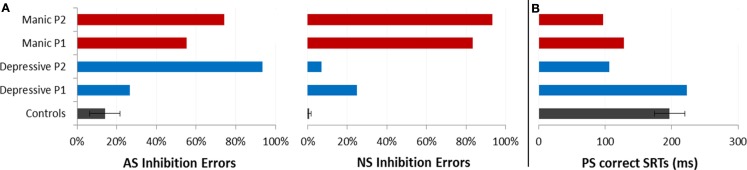
**Saccadic performance for each patient as a function of the two opposing illness phases (Depressive and Manic) and for controls (±CI)**. **(A)** Mean percentages of saccadic inhibition errors in antisaccades (AS) and nosaccades (NS). **(B)** Mean SRT (in milliseconds) in prosaccades (PS).

Saccadic performance is presented in Figure 2. We analyzed the inhibition errors, i.e., the saccades toward the cue in the NS and AS tasks. We also analyzed SRT for correct saccades for the two tasks requiring saccade execution, AS and PS.

#### Analysis of inhibition errors

We reported the inhibition error rate for the first saccade after cue onset. These errors only occurred for AS and NS, and are summarized in Table 2 and Figure 2A.

**Table 2:** **Saccadic inhibition error rates as a function of saccadic task (Antisaccade vs. Nosaccade), cue visual hemifield (Cue left vs. right), and illness phase (Depressive, Manic, Euthymic) for each patient (P1, P2) and for controls**.

	Task	Antisaccade		Nosaccade	
	Cue location	Left (%)	Right (%)	Left (%)	Right (%)
/	Controls	14	16	1	2
P1	Depressive	21	31	13	38
	Euthymic	43	53	14	38
	Manic	40	71	67	100
P2	Depressive	93	93	0	17
	Euthymic	100	93	14	0
	Manic	64	85	86	100

Table 2 shows that the two patients presented performance asymmetry related to the visual hemifield (cue position) in which the cue was displayed. Irrespectively of mood phase and saccade type, Patient 1 displayed weaker performance when the cue was flashed in the right hemifield, with more erroneous inhibition saccades in AS and NS tasks. This was also the case for Patient 2, except for AS in his depressive phase.

As shown in Figure 2, in the AS task, mean percentages of saccadic inhibition errors were very high compared to controls. In the NS task, mean percentages of saccadic inhibition errors varied dramatically according to mood phase. The number of NS inhibition errors was low in depressive as in euthymic periods (mean 17%) but very high in manic periods (88%). This pattern of results was observed for each patient (Table 2). For AS and NS, the 95% confidence interval (CI) for the percentage of inhibition errors was calculated using the controls’ results. Both patients had percentages of inhibition errors for AS and NS in theirs mood phases outside calculated CIs.

#### Analysis of correct saccades latencies

Because patients made a large number of AS errors, we carried out a more specific study of correct latencies for PS (Figure 2B). Patients displayed about 70% of correct PS saccades whatever their mood period. The incorrect saccades were mainly due to the fact that they made several hypometric (small) saccades to reach the correct target location.

We ran an item analysis. For both patients, mean correct SRTs were compared between the two illness phases. Paired *t*-test comparisons of mean SRTs showed a significant variation according to mood phase. Saccade latencies were significantly shorter in the manic phase compared to the depressive phase for each patient [P1: 128 vs. 223 ms, *t*(20) = 3.18; *p* < 0.005; P2: 97 vs. 106 ms, *t*(20) = 2.67, *p* < 0.05].

### Cortical excitability

Table 3 summarizes cortical excitability measures (RMT, baseline, intracortical effects for short and long ISI) obtained in the control group and for the two patients according to which hemisphere was stimulated (Left vs. Right), and mood phase for patients (Depressive, Euthymic, Manic). RMT and Baseline results were given for information. These measures were necessary to calculate modifications in baseline in the paired-pulse condition according to temporal interval between pulses (ISI, cf. Materials and Methods). We were therefore interested in ICI and facilitation processes. Measures in ISI conditions are expressed in baseline percentages, and are considered as measures of “intracortical excitability.” Result analysis focuses on intracortical excitability.

**Table 3:** **Resting motor threshold (RMT), baseline MEP amplitude, and intracortical effects for short and long ISI for each hemisphere (LH/RH) in controls and patients**.

		RMT (%)		Baseline (μV)		Short ISI (% change)		Long ISI (% change)	
		LH	RH	LH	RH	LH	RH	LH	RH
	Controls	50	51	2118	1832	−61.6	−73.7	57.0	66.5
P1	Depressive	46	36	449	1441	−59.2	55.4	−22.6	−6.4
	Euthymic	43	32	1660	1434	−22.8	−65.6	−11.6	−71.1
	Manic	46	34	663	1352	−28.0	0.6	60.3	−1.8
P2	Depressive	48	44	1600	394	−59.6	−53.1	0.6	94.6
	Euthymic	42	44	1520	1498	−26.5	−69.0	19.2	319
	Manic	42	48	328	630	−56.1	−55.5	69.9	−38.6

As shown in Table 3, the two patients display intracortical excitability changes compared to controls and according to mood phases, with some between-subject variability. To simplify data analysis and better understand the processes involved in these changes, the performances of both patients were merged (the two patients were considered as a group) and only the antagonist mood phases were considered (Depressive vs. Manic. Reminder: the euthymic phase was clinically not very clear).

As mentioned before, several studies have demonstrated that for ISI of less than 6 ms, the conditioning stimulus inhibits the test stimulus, whereas for ISI of between 6 and 100 ms, the conditioning stimulation facilitates the test stimulation.

**Figure 3:**
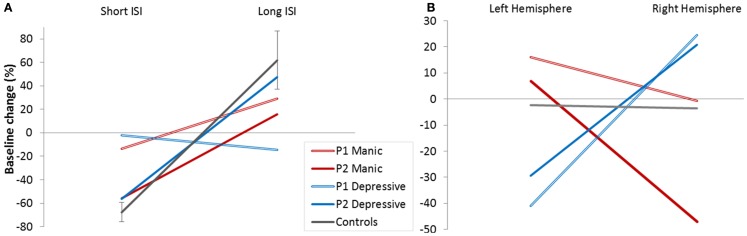
**Cortical excitability**. **(A)** Baseline modifications (%) in controls and patients (depressive/manic) according to Inter Stimulus Intervals (ISI short/long). **(B)** Baseline modifications (%) in controls and patients (depressive/manic) according to stimulated hemisphere.

We looked firstly at whether we found this effect of short (2 and 4 ms) and long ISI (10 and 15 ms) in patients and controls. Figure 3A represents mean baseline changes due to conditioning stimulus. In line with the literature, ICI was observed for short (2/4 ms) ISI, and facilitation (ICF) for long (10/15 ms) ISI (mean ICI = −68%, mean ICF = 62%) in controls.

We calculated the 95% CI in percentage of baseline change for long and short ISI calculated from the controls’ data. Patient’s results were compared to the calculated CI for long and short ISI. The pattern of excitability was reduced in patients compared to controls whatever their mood phases. Reduction was clear for the Patient 1 and for the Patient 2 for short ISI. For long ISI Patient P2 still had reduced percentage of baseline change in his manic phase. These results mean that a reduced global pattern of intracortical excitability could be indicative of the BD illness.

Figure 3B shows the global balance between inhibitory and facilitatory processes in left and right hemisphere. No global intracortical change was observed in controls (results did not differ from zero), meaning that the control group presented a good balance between intracortical excitability mechanisms and hemispheres.

Interestingly, this was not the case for both patients who showed a crossed interaction according to cerebral hemisphere. In the depressive state, ICI seems to predominate in the left hemisphere, and intracortical facilitation in the right hemisphere. The reverse pattern of global intracortical excitability was observed in the manic phase. Moreover, this interhemispheric asymmetry was observed for each patient (Table 3).

## Discussion

Despite the consensus for impaired saccadic performance in psychiatric disorders such as schizophrenia (Radant et al., 2007), there is very little research on mood disorders and eye movements. No study has aimed at investigating ocular movements in relation to the depressive vs. manic phases in the same patient (but, see Gooding et al., 2004). On the other hand, there is a relative consensus in literature that cortical excitability may be a biological correlate of disease in mood disorders (Wassermann et al., 2001; Bajbouj et al., 2006b; Levinson et al., 2010). Our study associated these two techniques with two bipolar patients to see if saccadic performance and cortical excitability measures could serve as a trait-marker and/or state-markers, and what the functional relationship between the two types of markers might be.

### Saccadic performance

Despite differences in the two patients’ medication, clinical histories (anamnesis) and intrinsic saccadic performances (Patient 1 displayed very rapid saccades), both presented two specific saccadic performances. Compared to control participants, patients’ inhibition errors in the AS task were numerous, whatever their mood phase, and this could possibly be a trait-marker of rapid cycling BD. Compared to both themselves and controls, patients’ inhibition errors in the NS task were numerous in the manic phase, suggesting that it could be a state-marker of the illness phase in rapid cycling BD. What emerges from PS latencies significantly distinguished the mood periods, with, notably, very short latencies in manic phases. These results suggest that different types of dysfunctions might well underlie inhibition errors in the manic phase.

### Cortical excitability

As for saccadic performance, we observed changes in patients’ cortical excitability. Compared to controls and irrespective of mood phase, patients displayed a lower “mean” intracortical excitability (with a high degree of interhemispheric and phase variability in measures), which could be a trait-marker of rapid cycling BD. Compared to themselves, patients displayed a crossed asymmetry of intracortical processes according to cerebral cortices and thymic phases, which could be a state-marker of rapid cycling BD.

Cortical excitability depends on the balance between inhibitory processes which suppress cortical activity, and excitatory ones which stimulate it (Nakamura et al., 1997). We wondered what the functional relationship between the imbalance of ICI and facilitation observed in patients and their deficits in saccadic inhibitions might be.

### Functional relationships between oculometric and intracortical markers?

#### Trait-markers

Both patients displayed a persistent inhibition deficit in AS, as well as a lower mean intracortical excitability, with a high degree of interhemispheric variability. Executing an appropriate AS depends on inhibitory and excitatory control, brought into play in neural circuitry involving a number of frontal structures (notably the prefrontal dorsolateral cortex and the frontal eye field), and subcortical structures (the basal ganglia, thalamus, superior colliculus), and their accurate intra and interhemispheric management. In other words, the execution of a correct AS requires the possession of efficient frontal-striatal-thalamus-SC GABAergic and glutamatergic networks (Munoz and Everling, 2004). This does not seem to be the case for these patients, as can be seen from their less efficient intracortical excitability (on average, compared to controls), as well as the strong interhemispheric variability of their inhibition (ICI) and facilitation (ICF) data. In short, the deficiency of inhibition processes in the AS task observed in the two patients, whatever their mood phase, could stem from the global dysfunction of intracortical excitability processes observed in these patients.

#### State-markers

The inhibition deficit in the NS task, observed during the manic phase in both patients, could depend on a more specific mood phase and hemisphere dependent GABAergic and/or Glutamatergic imbalance. Using a saccade paradigm similar to ours, Brown et al. (2006) compared fMRI activation patterns for PS, AS, and NS (called no-go trials) in humans. According to this research, inhibitory control is more dependent on the right hemisphere than on the left one. Patients’ data obtained in our study are consistent with this suggestion, with patients displaying fewer inhibition errors when the cue was flashed in the left hemifield (Table 2). If we observe mean right cortical excitability in patients, we can see that our two patients did not present any intracortical facilitation during manic phases (Figure 3B) but rather an ICI. The frontal-SC neural circuitry controlling ocular saccades is intricate and involves various subcortical structures as well as GABAergic and glutamatergic pathways. We should, however, note the existence of a direct top-down glutamatergic neural pathway from the frontal cortex to fixation neurons in the superior colliculus (Olivier et al., 2000; Munoz and Everling, 2004). So, if one assumes that the right hemisphere plays a major role in saccadic control, and more especially via the direct glutamatergic pathway on fixation neurons in the SC (fixation neuron activation must be maintained in the NS task despite the absence of a fixation dot), the right hemisphere glutamatergic deficit observed in patients during the manic phase could underlie the poor NS performance of patients in the manic phase.

### Limitations

This pilot and princeps follow-up study aimed to see whether it is possible to find psychophysical and neurophysiological trait- and state-markers in mood disorders, using a particular clinical paradigm (patients suffering from rapid cycling bipolar disorder) and two experimental tools (saccadic tasks and cortical excitability measured on the motor cortex via TMS pulses). The data obtained appears interesting and relevant, indicating that these two tools show changes in BD phases, with a possible functional coherence between them. However, this research does have limits. One major limit is the number of patients; the evaluation of only two patients is insufficient to allow any definite conclusions to be drawn. It should however, be noted that the prevalence of this psychiatric illness is low, and it is therefore difficult to form a significant group of rcBD patients. Secondly, patients were older than controls but despite the limitations due to the difference in ages, the observation of strong changes in NS performances and in cortical excitability asymmetries depending on the mood phase suggests that these effects are unrelated to age but to pathology. Moreover, it can be even more surprising to observe intracortical processes switches in older patients if we consider the reduction in cortical plasticity demonstrated by Freitas et al. (2011).

Another limit concerns the interpretation of results. On the one hand, GABAergic and glutamatergic functions were not directly estimated but indirectly inferred from MEP resulting from paired-pulse TMS applied to the motor cortex. On the other hand, the cerebral circuitry underlying ocular movements is very intricate and involves interacting GABAergic and glutamatergic pathways. Therefore, explaining patients’ saccadic performance by precise GABAergic and glutamatergic dysfunctions is, to say the least, a “delicate” theoretical exercise. However, postulating that an efficient saccadic control requires efficient GABAergic and glutamatergic neurotransmission is “reasonable.”

### Links with other studies

Several papers have tried to distinguish different processes in cognitive and motor inhibition (Nigg, 2000; Friedman and Miyake, 2004; Fournet et al., 2007). In his paper, Nigg (2000) opposed oculomotor inhibition (“intentional behavioral inhibition”) to different kinds of cognitive inhibition (“intentional cognitive resistance to interferences” or “intentional cognitive inhibition”). Our results suggest that oculomotor inhibition may involve different kinds of cognitive processes. As in the Stroop task, the AS task involves “interference control for behavioral response” and this type of control would thus be impaired in rapid cycling BD. On the other hand, as in the classical No-go task, the NS task involves “intentional motor inhibition” related to the suppression of motor response to distractors and this type of control would thus be impaired in the manic phase of rapid cycling BD throughout the DLPFC – Superior Colliculus – Frontal Eye Field network dependent on inhibitory and excitatory control (Alexander et al., 1990; Cummings, 1993). From this perspective, an immuno-histochemical study by Olivier et al. (2000) presented evidence for the presence of glutamatergic neurons in the cat superior colliculus, and confirmed the existence of these neurons in the rostral zone where SC fixation neurons SC project contralaterally.

Swann et al. (2009) wrote that pathological impulsivity in BD could be related to deficiencies in attention and response inhibition in more severe illness courses, therefore inhibition deficits could correspond to promising endophenotypes. According to Cherlyn et al. (2010), BD can be seen as complex disorders in synaptic neurotransmission particularly involving Glutamate and GABA neurotransmitters. Along with deficits in glutamatergic neurotransmission, hyperactivity of GABAergic neurotransmission is thought to be associated with schizophrenia and mania, as evidenced by the psychotomimetic effects of GABA_A_ receptor agonists (Tamminga et al., 1978; Hoehn-Saric, 1983). Our data are in agreement with these observations, more particularly if we observe right hemisphere changes. Indeed, we observed an association between NS inhibition errors in the manic phase and deficits in glutamatergic communication in the right hemisphere, which is the principal hemisphere for saccadic control.

To conclude, little is known about the categorization and understanding of mood disorders yet. In order to better understand the pathological mechanisms of these psychiatric disorders, identification of different types of illness markers, from genetic and chemical to neurophysiological and behavioral levels, appears to be an essential research approach. This study seems to indicate that oculometry (psychophysical) and cortical excitability (neurophysiological) can provide useful and coherent markers of bipolar disorders and their phases. However, these results need confirmation on an important number of patients with rapid cycling BD.

## Conflict of Interest Statement

The authors declare that the research was conducted in the absence of any commercial or financial relationships that could be construed as a potential conflict of interest.

## References

[B1] Alexander G. E. Crutcher M. D. Delong M. R. (1990). Basal ganglia-thalamocortical circuits: parallel substrates for motor, oculomotor, “prefrontal” and “limbic” functions. Prog. Brain Res. 85, 119–146 10.1016/S0079-6123(08)62678-3 2094891

[B2] Bajbouj M. Lang U. E. Niehaus L. Hellen F. E. Heuser I. Neu P. (2006a). Effects of right unilateral electroconvulsive therapy on motor cortical excitability in depressive patients. J. Psychiatr. Res. 40, 322–327 10.1016/j.jpsychires.2005.07.002 16137698

[B3] Bajbouj M. Lisanby S. H. Lang U. E. Danker-Hopfe H. Heuser I. Neu P. (2006b). Evidence for impaired cortical inhibition in patients with unipolar major depression. Biol. Psychiatry 59, 395–400 10.1016/j.biopsych.2005.07.036 16197927

[B4] Baxter L. R. Jr. Schwartz J. M. Phelps M. E. Mazziotta J. C. Guze B. H. Selin C. E. (1989). Reduction of prefrontal cortex glucose metabolism common to three types of depression. Arch. Gen. Psychiatry 46, 243–250 10.1001/archpsyc.1989.01810030049007 2784046

[B5] Brown M. R. Goltz H. C. Vilis T. Ford K. A. Everling S. (2006). Inhibition and generation of saccades: rapid event-related fMRI of prosaccades, antisaccades, and nogo trials. Neuroimage 33, 644–659 10.1016/j.neuroimage.2006.07.002 16949303

[B6] Campbell S. MacQueen G. (2006). An update on regional brain volume differences associated with mood disorders. Curr. Opin. Psychiatry 19, 25–33 10.1097/01.yco.0000194371.47685.f2 16612175

[B7] Cherlyn S. Y. Woon P. S. Liu J. J. Ong W. Y. Tsai G. C. Sim K. (2010). Genetic association studies of glutamate, GABA and related genes in schizophrenia and bipolar disorder: a decade of advance. Neurosci. Biobehav. Rev. 34, 958–977 10.1016/j.neubiorev.2010.01.002 20060416

[B8] Chroni E. Lekka N. P. Argyriou A. A. Polychronopoulos P. Beratis S. (2008). Persistent suppression of postexercise facilitation of motor evoked potentials during alternate phases of bipolar disorder. J. Clin. Neurophysiol. 25, 115–118 10.1097/WNP.0b013e31816ef739 18340269

[B9] Crevits L. Van Den Abbeele D. Audenaert K. Goethals M. Dierick M. (2005). Effect of repetitive transcranial magnetic stimulation on saccades in depression: a pilot study. Psychiatry Res. 135, 113–119 10.1016/j.psychres.2003.10.008 15919118

[B10] Cummings J. L. (1993). Frontal-subcortical circuits and human behavior. Arch. Neurol. 50, 873–880 10.1001/archneur.1993.00540080076020 8352676

[B11] Davidson J. R. Meltzer-Brody S. E. (1999). The underrecognition and undertreatment of depression: what is the breadth and depth of the problem? J. Clin. Psychiatry 60(Suppl. 7), 4–9; discussion 10–11. 10.4088/JCP.v60n0805 10326869

[B12] Fournet N. Mosca C. Moreaud O. (2007). Deficits in inhibitory processes in normal aging and patients with Alzheimer’s disease: a review. Psychol. Neuropsychiatr. Vieil. 5, 281–294 18048106

[B13] Freitas C. Perez J. Knobel M. Tormos J. M. Oberman L. Eldaief M. (2011). Changes in cortical plasticity across the lifespan. Front. Aging Neurosci. 3:5 10.3389/fnagi.2011.00005 21519394 PMC3079175

[B14] Friedman N. P. Miyake A. (2004). The relations among inhibition and interference control functions: a latent-variable analysis. J. Exp. Psychol. Gen. 133, 101–135 10.1037/0096-3445.133.1.101 14979754

[B15] Fukushima J. Fukushima K. Chiba T. Tanaka S. Yamashita I. Kato M. (1988). Disturbances of voluntary control of saccadic eye movements in schizophrenic patients. Biol. Psychiatry 23, 670–677 10.1016/0006-3223(88)90050-9 3370264

[B16] Fukushima J. Fukushima K. Morita N. Yamashita I. (1990a). Further analysis of the control of voluntary saccadic eye movements in schizophrenic patients. Biol. Psychiatry 28, 943–958 10.1016/0006-3223(90)90060-F 2275952

[B17] Fukushima J. Morita N. Fukushima K. Chiba T. Tanaka S. Yamashita I. (1990b). Voluntary control of saccadic eye movements in patients with schizophrenic and affective disorders. J. Psychiatr. Res. 24, 9–24 10.1016/0022-3956(90)90021-H 2366215

[B18] Gooding D. C. Basso M. A. (2008). The tell-tale tasks: a review of saccadic research in psychiatric patient populations. Brain Cogn. 68, 371–390 10.1016/j.bandc.2008.08.024 18950927 PMC2755089

[B19] Gooding D. C. Mohapatra L. Shea H. B. (2004). Temporal stability of saccadic task performance in schizophrenia and bipolar patients. Psychol. Med. 34, 921–932 10.1017/S003329170300165X 15500312

[B20] Guyader N. Malsert J. Marendaz C. (2010). Having to identify a target reduces latencies in prosaccades but not in antisaccades. Psychol. Res. 74, 12–20 10.1007/s00426-008-0218-7 19104829

[B21] Hallett P. E. (1978). Primary and secondary saccades to goals defined by instructions. Vision Res. 18, 1279–1296 10.1016/0042-6989(78)90218-3 726270

[B22] Harris M. S. Reilly J. L. Thase M. E. Keshavan M. S. Sweeney J. A. (2009). Response suppression deficits in treatment-naive first-episode patients with schizophrenia, psychotic bipolar disorder and psychotic major depression. Psychiatry Res 170, 150–156 10.1016/j.psychres.2008.10.031 19906441 PMC2792232

[B23] Hoehn-Saric R. (1983). Effects of THIP on chronic anxiety. Psychopharmacology (Berl.) 80, 338–341 10.1007/BF00432116 6414002

[B24] Kapogiannis D. Wassermann E. M. (2008). Transcranial magnetic stimulation in clinical pharmacology. Cent. Nerv. Syst. Agents Med. Chem. 8, 234–240 10.2174/187152408786848076 19122782 PMC2613312

[B25] Kujirai T. Caramia M. D. Rothwell J. C. Day B. L. Thompson P. D. Ferbert A. (1993). Corticocortical inhibition in human motor cortex. J. Physiol. (Lond.) 471, 501–519 8120818 10.1113/jphysiol.1993.sp019912 PMC1143973

[B26] Levinson A. J. Fitzgerald P. B. Favalli G. Blumberger D. M. Daigle M. Daskalakis Z. J. (2010). Evidence of cortical inhibitory deficits in major depressive disorder. Biol. Psychiatry 67, 458–464 10.1016/j.biopsych.2009.09.025 19922906

[B27] Levinson A. J. Young L. T. Fitzgerald P. B. Daskalakis Z. J. (2007). Cortical inhibitory dysfunction in bipolar disorder: a study using transcranial magnetic stimulation. J. Clin. Psychopharmacol. 27, 493–497 10.1097/jcp.0b013e31814ce524 17873683

[B28] Maeda F. Keenan J. P. Pascual-Leone A. (2000). Interhemispheric asymmetry of motor cortical excitability in major depression as measured by transcranial magnetic stimulation. Br. J. Psychiatry 177, 169–173 10.1192/bjp.177.2.169 11026958

[B29] Markram H. Toledo-Rodriguez M. Wang Y. Gupta A. Silberberg G. Wu C. (2004). Interneurons of the neocortical inhibitory system. Nat. Rev. Neurosci. 5, 793–807 10.1038/nrn1519 15378039

[B30] McDowell J. E. Brown G. G. Paulus M. Martinez A. Stewart S. E. Dubowitz D. J. (2002). Neural correlates of refixation saccades and antisaccades in normal and schizophrenia subjects. Biol. Psychiatry 51, 216–223 10.1016/S0006-3223(01)01204-5 11839364

[B31] Montgomery S. A. Asberg M. (1979). A new depression scale designed to be sensitive to change. Br. J. Psychiatry 134, 382–389 10.1192/bjp.134.4.382 444788

[B32] Munoz D. P. Everling S. (2004). Look away: the anti-saccade task and the voluntary control of eye movement. Nat. Rev. Neurosci. 5, 218–228 10.1038/nrn1345 14976521

[B33] Nakamura H. Kitagawa H. Kawaguchi Y. Tsuji H. (1997). Intracortical facilitation and inhibition after transcranial magnetic stimulation in conscious humans. J. Physiol. (Lond.) 498(Pt 3), 817–823 9051592 10.1113/jphysiol.1997.sp021905 PMC1159197

[B34] Nigg J. T. (2000). On inhibition/disinhibition in developmental psychopathology: views from cognitive and personality psychology and a working inhibition taxonomy. Psychol. Bull. 126, 220–246 10.1037/0033-2909.126.2.220 10748641

[B35] Olivier E. Corvisier J. Pauluis Q. Hardy O. (2000). Evidence for glutamatergic tectotectal neurons in the cat superior colliculus: a comparison with GABAergic tectotectal neurons. Eur. J. Neurosci. 12, 2354–2366 10.1046/j.1460-9568.2000.00132.x 10947814

[B36] Radant A. D. Dobie D. J. Calkins M. E. Olincy A. Braff D. L. Cadenhead K. S. (2007). Successful multi-site measurement of antisaccade performance deficits in schizophrenia. Schizophr. Res. 89, 320–329 10.1016/j.schres.2006.08.010 17023145

[B37] Samii A. Wassermann E. M. Ikoma K. Mercuri B. George M. S. O’Fallon A. (1996). Decreased postexercise facilitation of motor evoked potentials in patients with chronic fatigue syndrome or depression. Neurology 47, 1410–1414 10.1212/WNL.47.6.1410 8960719

[B38] Shajahan P. M. Glabus M. F. Gooding P. A. Shah P. J. Ebmeier K. P. (1999). Reduced cortical excitability in depression. Impaired post-exercise motor facilitation with transcranial magnetic stimulation. Br. J. Psychiatry 174, 449–454 10.1192/bjp.174.5.449 10616615

[B39] Swann A. C. Lijffijt M. Lane S. D. Steinberg J. L. Moeller F. G. (2009). Severity of bipolar disorder is associated with impairment of response inhibition. J. Affect. Disord. 116, 30–36 10.1016/j.jad.2008.10.022 19038460 PMC2693289

[B40] Tamminga C. A. Crayton J. W. Chase T. N. (1978). Muscimol: GABA agonist therapy in schizophrenia. Am. J. Psychiatry 135, 746–747 350058 10.1176/ajp.135.6.746

[B41] Wassermann E. M. (1998). Risk and safety of repetitive transcranial magnetic stimulation: report and suggested guidelines from the International Workshop on the Safety of Repetitive Transcranial Magnetic Stimulation, June 5–7, 1996. Electroencephalogr. Clin. Neurophysiol. 108, 1–16 10.1016/S0168-5597(97)00096-8 9474057

[B42] Wassermann E. M. Greenberg B. D. Nguyen M. B. Murphy D. L. (2001). Motor cortex excitability correlates with an anxiety-related personality trait. Biol. Psychiatry 50, 377–382 10.1016/S0006-3223(01)01210-0 11543742

[B43] World Health Organization (WHO) (2003). The World Health Report -- Shaping the Future. Geneva: WHO

[B44] Young R. C. Biggs J. T. Ziegler V. E. Meyer D. A. (1978). A rating scale for mania: reliability, validity and sensitivity. Br. J. Psychiatry 133, 429–435 10.1192/bjp.133.5.429 728692

[B45] Ziemann U. Chen R. Cohen L. G. Hallett M. (1998). Dextromethorphan decreases the excitability of the human motor cortex. Neurology 51, 1320–1324 10.1212/WNL.51.6.1771-a 9818853

[B46] Ziemann U. Rothwell J. C. Ridding M. C. (1996). Interaction between intracortical inhibition and facilitation in human motor cortex. J. Physiol. (Lond.) 496(Pt 3), 873–881 8930851 10.1113/jphysiol.1996.sp021734 PMC1160871

